# Regression of fibrosis in paediatric autoimmune hepatitis: morphometric assessment of fibrosis versus semiquantiatative methods

**DOI:** 10.1186/1755-1536-2-2

**Published:** 2009-04-02

**Authors:** Ahmed F Abdalla, Khaled R Zalata, Abeer F Ismail, Gamal Shiha, Mohamed Attiya, Ahmed Abo-Alyazeed

**Affiliations:** 1 Department of Paediatrics, Hepatology & Gastroenterology Unit, Faculty of Medicine, Mansoura University, Mansoura, Egypt; 2 Department of Pathology, Faculty of Medicine, Mansoura University, Mansoura, Egypt; 3 Department of Internal Medicine, Hepatology & Gastroenterology Unit, Faculty of Medicine, Mansoura University, Mansoura, Egypt; 4 Department of Public Health, Faculty of Medicine, Mansoura University, Mansoura, Egypt

## Abstract

**Background:**

Regression of hepatic fibrosis in patients with autoimmune hepatitis (AIH) has been described in response to immunosuppressive therapy. These studies, however, besides being few in number, were conducted on adult populations. Our aim was to assess the regression of hepatic fibrosis, using morphometric assessment of fibrosis versus semi-quantitative methods, in children with AIH who achieved clinical and biochemical remission. Thirteen patients who achieved clinical and biochemical remission were included in the study, out of 62 children with AIH. Repeat biopsy was performed after 6 to 12 months of clinical and biochemical remission. Morphometric assessment of fibrosis was performed and correlated with METAVIR and Ishak semi-quantitative scores.

**Results:**

The study group included eight male and five female patients. The median age at presentation was 4 years (range 2 to 12 years). The mean duration of treatment was 22 ± 7.3 months, and the mean interval between biopsies was 26.2 ± 6.5 months. Following therapy, there was significant reduction in aspartate aminotransferase, ALT and IgG levels as well as improvement of necroinflammation. The mean fibrosis scores were significantly decreased from 4.5 ± 1.19 and 2.9 ± 0.7 before therapy to 2.7 ± 1.16 and 2 ± 0.8 after treatment as assessed by Ishak and METAVIR scores, respectively (P = 0.001 and 0.004). The mean morphometric assessment of fibrosis before treatment was 20% ± 9.7 and following therapy it decreased to 5.6% ± 3.9 (P = 0.000).

**Conclusion:**

Significant regression of fibrosis in paediatric AIH could occur with current therapeutic regimens. Morphometric assessment of fibrosis is more sensitive than semi-quantitative methods to identify changes in fibrosis.

## Background

Autoimmune hepatitis (AIH) remains an enigmatic condition that affects children of all ages. It accounts for 2% to 5% of paediatric liver disease; however, the disease process in children appears to be more severe at presentation than commonly seen in adults, perhaps because of delay in diagnosis. Over 50% of children have cirrhosis at accession, and the disease commonly has an aggressive course [1].

AIH reflects a complex interaction between triggering factors, autoantigens, genetic predisposition and immunoregulatory networks [2]. Currently, the basic treatment of AIH is prednisone and/or azathioprine. Treatment aims at obtaining full remission not only at the clinical and biochemical levels but also at the histological level. Remission connotes disappearance of symptoms, lack of biochemical manifestations of inflammation (aspartate aminotransferase (AST) level should not be more than twice the upper normal limit, globulin levels should be normal) with the histological findings showing lack of activity or minimal activity of the process [3,4].

Cirrhosis is an end-stage process of chronic progressive scarring inflammation produced by many causes. Once cirrhosis is established, it had been considered to be irreversible. When complications of cirrhosis such as ascites, severe encephalopathy and jaundice with variceal bleeding develop, the survival of cirrhotic patients becomes short and lethality is unavoidable. However, reports about a variety of liver disease states suggest that even established cirrhosis might be reversible with certain therapeutic regimens. Regression of fibrosis has followed phlebotomy for haemochromatosis [5], relief of chronic biliary obstruction [6] and bone marrow transplantation for thalassemia [7]. Reports suggest improvement of cirrhosis in patients with primary biliary cirrhosis treated with ursodeoxycholic acid and methotrexate [8], and also in patients with Wilson's disease treated with penicillamine [9]. Recently, there has been a documented regression of fibrosis in several patients of chronic hepatitis B and C treated with antiviral agents [10-13]. These observations in humans have been supported by murine models of hepatic injury in which biliary fibrosis has decreased after biliary decompression [14] and rabbit models in which liver fibrosis has regressed after treatment of schistosomiasis [15].

Few reports are available on the regression of hepatic fibrosis in patients with autoimmune hepatitis in response to immunosuppressive therapy [16,17]. All these studies, however, were conducted on adult populations and only one, so far, was conducted on paediatric patients [18]. Morphometric assessment of fibrosis by image analysis is becoming more sensitive and accurate than semi-quantitative methods for the assessment of hepatic fibrosis [19,20].

The aim of this study was to assess the possible regression of hepatic fibrosis, using the morphometric assessment of fibrosis versus semi-quantitative methods, in children with AIH treated with prednisone and/or azathioprine who achieved clinical and biochemical remission.

## Methods

### Study population

Thirteen patients (eight males and five females) with AIH, who achieved clinical and biochemical remission in response to treatment with prednisone and/or azathioprine, were included out of 62 patients with AIH who were seen at Mansoura University Children's Hospital, Paediatric GI and Hepatology Unit between 1999 and 2007. The remaining 49 patients were not included because they either did not have significant fibrosis (≤ 2/6, Ishak score) (0 or F1, METAVIR) at presentation, not enough time had elapsed for post-remission biopsy, had relapses, or failed to give consent.

Autoimmune hepatitis was diagnosed if the patient had chronically elevated serum aminotransferase levels, hypergammaglobulinaemia, positive autoantibodies (antinuclear antibody (ANA), anti-smooth muscle antibody (SMA), anti liver-kidney microsomal (LKM) antibody type 1), histopathological features compatible with autoimmune hepatitis on liver biopsy, and no evidence of viral infection (hepatitis B and C), metabolic (Wilson's disease, α1-antitrypsin deficiency, hemochromatosis) or drug-induced liver disease. We applied the revised scoring system for diagnosis of autoimmune hepatitis with interpretation of aggregated score; Definite AIH >15 and probable AIH 10 to 15 [21]. Included children were divided into two groups: Type 1 (ANA/SMA positive) and Type 2 (LKM-1 positive).

### Treatment regimen and outcome

After diagnosis, treatment was initiated by prednisone; 2 mg/kg/day (up to 60 mg) for 2 weeks, then gradually tapered over 6 to 8 weeks, guided by the clinical and biochemical responses to a maintenance dose of 0.1 to 0.2 mg/kg/day or 5 mg daily, plus azathioprine 1 to 2 mg/kg/day (unless it is contraindicated due to cytopenia). Remission was defined as disappearance of symptoms with no flares, improvement of aminotransferase to less than twice normal (maintained for at least 1 to 2 years), restoration of serum bilirubin and γ globulin level to normal, and histological findings showing lack of activity or minimal activity. The included children underwent a second biopsy after 1 to 2 years of remission.

### Histological assessment

After obtaining an informed and written consent, 26 liver biopsies from these 13 children were obtained (pre and post-treatment) by blind percutaneous biopsy (Bard gun biopsy with Tru-cut needle, gauge 16). The length of liver biopsies was 1.5 cm or more; the number of portal tracts was six or more. The cores were fixed in 10% formaldehyde-saline and processed according to routine histological techniques, until paraffin embedding.

For routine pathological diagnosis, sections were stained with haematoxylin and eosin, Masson's trichrome, and periodic acid shift before and after diastase digestion. Histological features were semi-quantitatively graded according to the Ishak modified Knodell Score [22] and METAVIR system [23]. The histological evaluation was performed systemically by one hepatopathologist (KZ) who was blinded to the clinical and serological data.

**Figure 1:**
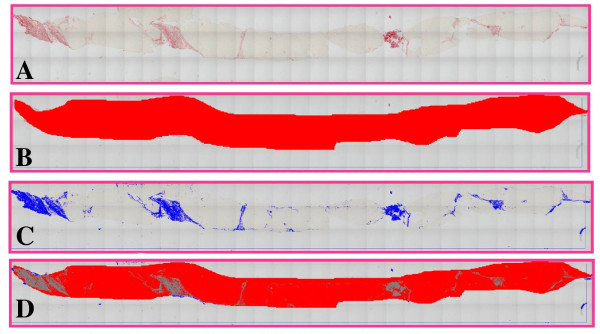
**Morphometric assessment of liver fibrosis in post-treatment biopsy using mosaic picture**. a) Post-treatment mosaic picture of 93 microscopic fields (Sirus redx100). b) Delineation of the whole liver core (mosaic picture). c) Computer-assisted marking of fibrosis in binary image. d) Combined marking of fibrosis and the whole core at the same time.

The morphometric assessment of liver fibrosis was performed by the fully automated Leica image processor with automated stage and Leica Quin software 2004. The liver biopsy slide, stained with Sirus red, was placed on the x-y motorized stage of Leica microscope. At a magnification of ×10, automated sequential digitalized images were taken and stored, then a mosaic picture was created including all the images with minimal field overlapping. This enables fibrosis assessment of the entire core at the same time. After interactive threshold, the image was converted into a binary image. Artefacts created during slide preparation were eliminated by both automatic and interactive procedures (Figure 1a to 1d). The area of the liver parenchyma was considered the reference area, then the fractional surface occupied by fibrosis was measured within the above-defined area.

### Statistical analysis

The statistical analysis of data was done using SPSS program statistical package for social science version 10. The description of the data was performed in the form of mean (±) standard deviation for quantitative data and frequency and proportion for qualitative data. Non-parametric data were tested by Kolmogrov-Smirnov test and was represented as median (minimum-maximum). Pearson correlation was applied to test the association between morphometry, Ishak and METAVIR.

The analysis of the data was done to test statistically significant differences between groups. Paired sample t-test was used to compare one group at different times, and Wilcoxon test was used to compare non-parametric data at different times. P is significant if ≤ or = 0.05 at confidence interval 95%.

## Results

### Demographic and biochemical results

The median age at presentation was 4 years (range 2 to 12 years). Ten children were classified as Type 1 while the remaining three were Type 2. At presentation, patients had elevated alanine aminotransferase (ALT) levels (median = 243 IU/l, range 19 to 870 IU/l), AST levels (median = 170 IU/l, range 19 to 800 IU/l), and immunoglobulin G (IgG) levels (median = 27 mg/dl, range 13 to 48 mg/dl) (Table 1). The median albumin level was 3.7 g/dl (range 2.9 to 5.2 g/dl), and the median platelet count was 189,000 cells/mm3 (range 78,000 cells/mm^3 ^to 400,000 cells/mm^3^). Bilirubin level was 4.5 mg/dl (range 0.5 to 17.0 mg/dl). Their prothrombin times ranged from 11 to 17 seconds. All the included patients were classified according to the revised IAHG scoring system before treatment as definite AIH, with score range from 16 to 20. There was no post-treatment reassessment, as all the included children were definite AIH before treatment (score>15).

**Table 1:** Laboratory investigations of the studied group before and after treatment

	Before treatment			After treatment		
	
Serial	ALT	AST	IgG	ALT	AST	IgG
1	673	600	21.8	10	45	12
2	540	560	13.3	17	47	14.5
3	585	412	48	30	36	18
4	83	85	17.3	30	22	14.4
5	50	95	28	40	45	12.6
6	870	800	23	52	53	16.3
7	156	170	32	34	49	14.8
8	314	100	27	102	91	18
9	39	19	28	26	15	14.4
10	35	40	17.7	18	22	17
11	19	26	20.6	20	24	15
12	243	230	27.8	40	48	14.4
13	502	671	30.5	19	33	16
Mean	316.0769	292.9231	25.7692	33.6923	40.7692	15.1846
Median	243	170	27	30	45	14.8
Std. Deviation	287.4275	278.2117	8.7248	23.5527	19.5796	1.8343
Minimum	19	19	13.3	10	15	12
Maximum	870	800	48	102	91	18

### Duration of therapy and follow up

The follow up period varied from 2 years to 7.5 years (mean = 4.07 ± 2.9 years). The mean duration of treatment was 22 ± 7.3 months. The mean interval between diagnostic biopsies and those made to determine discontinuation of treatment was 26.2 ± 6.5 months. The mean time elapsed from clinical and biochemical remission till liver biopsy 17.8 ± 8.5 months. Following therapy, there was a significant reduction in AST, ALT and IgG levels as shown in Table 1.

### Histological assessment

The mean necroinflammation score before treatment was 8.07 ± 3.5 and 2.2 ± 0.9 as assessed by Ishak and METAVIR scores respectively. Following treatment, there was a significant reduction in necroinflammation (4.6 ± 2.4 and 1.07 ± 0.6 as assessed by Ishak and METAVIR scores respectively), P = 0.004 and 0.001 (Table 2).

**Table 2:** Activity scores of the studied group before and after treatment

	Activity before treatment		Activity after treatment	
Serial	Ishak	METAVIR	Ishak	METAVIR

1	4	1	4	1
2	9	3	1	0
3	11	3	6	1
4	5	1	6	1
5	8	2	5	1
6	8	2	8	2
7	13	3	6	2
8	9	3	8	2
9	10	3	6	1
10	3	1	3	1
11	2	1	0	0
12	13	3	4	1
13	10	3	3	1
Mean	8.0769	2.2308	4.6154	1.0769
Median	9.0000	3.0000	5.0000	1.0000
Std. Deviation	3.5931	.9268	2.4337	.6405
Minimum	2.00	1.00	.00	.00
Maximum	13.00	3.00	8.00	2.00

The stage of fibrosis was assessed semi-quantitatively by both Ishak and METAVIR scores. The mean fibrosis score before treatment was 4.5 ± 1.19 and 2.9 ± 0.7 respectively. Following treatment, it decreased to 2.7 ± 1.16 and 2 ± 0.8. Such reduction was statistically significant (P = 0.001 and 0.004 for Ishak and METAVIR scores, respectively). In three children the METAVIR score remained unchanged (Table 3).

**Table 3:** Fibrosis scores of the studied group before and after treatment (Ishak, METAVIR, and Morphometry)

SERIAL	Before Fibrosis			After Fibrosis		
	Ishak	METAVIR	Morphometry%	Ishak	METAVIR	Morphometry%
1	3	2	9	3	2	3
2	5	3	18	1	1	2
3	5	3	36.3	3	2	8.1
4	5	3	27	4	3	11
5	4	3	15	1	1	2
6	3	2	13	4	3	10
7	5	3	25	4	3	1.1
8	6	4	30	4	3	12
9	5	3	32	3	2	10
10	3	2	7.9	2	1	3.4
11	3	2	5.3	1	1	1.8
12	6	4	20	3	2	5
13	6	4	21.8	3	2	3.6
Mean	4.5385	2.9231	20.0231	2.7692	2	5.6154
Median	5	3	20	3	2	3.6
Standard deviation	1.1983	0.7596	9.7827	1.1658	0.8165	3.9964
Minimum	3	2	5.3	1	1	1.1
Maximum	6	4	36.3	4	3	12

The mean morphometric assessment of fibrosis before treatment was 20% ± 9.7% and following therapy it decreased to 5.6% ± 3.9% (P = 0.000). In the three children whose METAVIR score remained unchanged, however, morphometry revealed a significant reduction in the degree of fibrosis (P < 0.001). On pre-treatment liver biopsies, Pearson correlation test revealed good correlation between Ishak and morphometry (correlation coefficient: 0.757, P < 0.01), and between METAVIR and morphometry (correlation coefficient: 0.635, P < 0.05). Furthermore, on post-treatment liver biopsies, there was a good correlation between Ishak and morphometry (correlation coefficient: 0.643, P < 0.05), and between METAVIR and morphometry (correlation coefficient: 0.636, P < 0.05) as well.

## Discussion

Hepatic fibrosis is the common end point for most types of chronic liver injury. It is usually considered to be an irreversible process, especially when there is evidence of cirrhosis [24]. Recent data have challenged this belief by showing that hepatic fibrosis is a dynamic process involving an imbalance between the deposition and the degradation of fibrillar collagens and other extracellular matrix proteins [25]. Hepatic fibrosis is amenable to intervention by removing the insult and stopping the persistent inflammatory stimuli [26].

We studied thirteen patients with documented cirrhosis or extensive fibrosis at the time of diagnosis of AIH in whom clinical and biochemical remission was achieved by prednisone and/or azathioprine therapy, with significant reduction in the necroinflammatory injury in post-treatment liver biopsies. We discovered a significant regression of fibrosis in these patients after 6 to 12 months of clinical and biochemical remission.

Previous studies have demonstrated that hepatic inflammation stimulates perivascular hepatic stellate cells by cytokines, such as transforming growth factor β1 (TGF-b), These activated stellate cells are transformed into myofibroblasts; they proliferate, migrate, and degrade the normal extracellular matrix and replace it with fibril-forming collagens types I and III [27]. The matrix proteins accumulate because tissue inhibitors retard the counteractive degradative actions of matrix metalloproteinases [28]. Suppression of inflammatory activity promotes disappearance of the metalloproteinase inhibitors, degradation of the fibrotic liver matrix by unrestricted metalloproteinases, and apoptosis of the hepatic stellate cells [29,30]. Our findings suggest that treatment with prednisone and/or azathioprine may inhibit the inciting stimulus for fibrogenesis and facilitate these anti-fibrotic actions in AIH by suppressing liver inflammation.

On the other hand, a glucocorticoid response element has been described in the human TGF-b1 gene promoter which may inhibit its expression [31]. Thus, corticosteroids may impair activation of TGF-b [32], alter its binding characteristics to matrix sites [33] and affect the ligation of TGF-b activator protein to the TGF-b element [34]. These putative anti-fibrotic actions of corticosteroids may complement their known anti-inflammatory effects to retard fibrogenesis and favour the counter-regulatory mechanisms of fibrinolysis [35,36].

Regression of hepatic fibrosis in AIH has been reported in adult patients [16,17,37-39], and there is one report on regression of hepatic fibrosis in paediatric cases of AIH [18]. In all these studies the assessment of fibrosis was done only semi-quantitatively by applying Ishak score. In our study we assessed hepatic fibrosis by semi-quantitative methods applying both Ishak and METAVIR scores and by a quantitative assay, morphometry. Regression of fibrosis was evident by both methods; however, by morphometry reduction of fibrosis was more significant. Moreover, cases in which no significant reduction of fibrosis was assessed by the semi-quantitative methods, morphometry could show a significant change. Ferreira et al found no change in fibrosis score in 25% of their patients but in one patient incomplete cirrhosis developed into complete cirrhosis [18]. In 70% of their patients there was reduction in fibrosis; however, the meantime elapsed from remission to liver biopsy was longer (4.1 ± 1.5 years) than in our study (17.8 ± 8.5 months) [18]. To the best of our knowledge, this is the first time that fibrosis reversibility has been assessed by the morphometric method in paediatric patients. Our findings suggest that quantitative assessment of fibrosis may be more sensitive in assessing regression of fibrosis following treatment of AIH. It has the benefit of avoiding the inter-observer variation in the assessment of fibrosis by semi-quantitative methods. Also, morphometry can assess the actual amount of fibrosis and not only its pattern, as in semi-quantitative methods. Moreover, application of morphometry on a mosaic picture of the entire core enabled the assessment of the portal as well the parenchymal fibrosis at the same time.

## Conclusion

Hepatic fibrosis and early cirrhosis are amenable to regression in patients with AIH achieving clinical and biochemical remissions in response to treatment. Early inclusion of prednisone and/or azathioprine might have great benefits on regression of fibrosis.

Morphometry is a more sensitive method to assess hepatic fibrosis regression in comparison with semi-quantitative methods (Ishak and METAVIR). Application of mosaic technology enables image analysis of the entire core without selection bias of certain areas.

## Abbreviations

AIH: Autoimmune Hepatitis; ALT: Alanine Aminotransferase; ANA: Anti-nuclear Antibody; AST: Aspartate Aminotransferase; IgG: Immunoglobulin G; LKM: anti-liver-kidney Microsomal: SAM: Anti-smooth Muscle Antibody; TGF-b: Transforming Growth Factor Beta.

## Competing interests

The authors declare that they have no competing interests.

## Authors' contributions

AA conceived of the study and participated in its design and coordination and helped to draft the manuscript. KZ carried out the histological and morphometric studies, participated in study design and helped to draft the manuscript. AI participated in the acquisition and interpretation of data and participated in drafting the manuscript. GS and MA were involved in revising the manuscript critically for important intellectual content. AAA participated in the design of the study and performed the statistical analysis. All authors read and approved the final manuscript.
